# Beyond fifth disease: atypical parvovirus B19 neurological syndromes in adults—first series from the West Bank

**DOI:** 10.1093/omcr/omag035

**Published:** 2026-04-14

**Authors:** Omar Marouf, Mohammed AbuBaha, Hossam Salameh, Wael Hashem, Hasan Khalili, Bashar Yaghi, Bara AbuBaha, Hatem M Taha

**Affiliations:** Department of Internal Medicine, Palestine Medical Complex, Al Ma’ahed street, Ramallah 601, Palestine; Department of Medicine, An-Najah National University, Rafidia street, Nablus P400, Palestine; Department of Medicine, An-Najah National University, Rafidia street, Nablus P400, Palestine; Department of Medicine, An-Najah National University, Rafidia street, Nablus P400, Palestine; Department of Medicine, An-Najah National University, Rafidia street, Nablus P400, Palestine; Department of Internal Medicine, Palestine Medical Complex, Al Ma’ahed street, Ramallah 601, Palestine; Department of Medicine, An-Najah National University, Rafidia street, Nablus P400, Palestine; Head of Medical Departments, Palestine Medical Complex, Al Ma’ahed street, Ramallah 601, Palestine; Teaching Faculty, Faculty of Medicine and Health Sciences, An-Najah National University, Rafidia street, Nablus P400, Palestine

**Keywords:** Parvovirus B19 infection, Central nervous system infections, atypical

## Abstract

Parvovirus B-19 (B19-PV) infection is usually a self-limiting illness that occurs in children, although there have been reported cases affecting older people and presenting with a wide range of symptoms, especially neurological ones. The diagnosis can be tricky and can cost time; therefore, knowledge of this infectious etiology and how it manifests can aid physicians in prompt and timely diagnosis, as time is vital in such cases in order to preserve organ functions and prevent life-threatening complications. Herein, we report Parvovirus B19-associated clinical features and workup in a series of five patients with laboratory-confirmed B19-PV from 2024 to 2025, which, to our knowledge, is the first case series in the West Bank of Palestine. The mean age of our population was 60.5 years, with a sex ratio (male: female) of 3:2, though of note, males were all above 60 and all females were under 50, mainly presenting with neurological manifestations.

## Introduction

B19 Parvovirus (B19-PV) belongs to the erythrovirus genus in the family of *Parvoviridae* [1]. Considered to be a relatively newly identified cause of erythema infectiosum (fifth disease), as it was first described by Anderson et al. in 1983 [2]. Generally affecting children evidenced by Douvoyiannis et al. in their review of 81 cases [3], which contrasts with the findings in our series of patients, where it was dominated by elderly and middle-aged individuals.

Douvoyiannis et al. reported neurological manifestations in 81% of their patients; notably, 39 cases developed encephalopathy, encephalitis, and/or meningoencephalitis, 14 of whom had altered immunity (36%), which showcases the seriousness and rarity of such clinical manifestations [3].

Confirmatory diagnostic modalities include the detection of specific antibodies against B19-PV from cerebrospinal fluid (CSF) or serum samples, or through B19-PV DNA on Polymerase Chain Reaction (PCR) testing [4].

Data on B19-PV is limited in the adult population. Knowledge and awareness of B19-PV infection should be attained and considered, especially in cases with atypical neurological complaints and an uncertain diagnostic picture, as this may prevent catastrophic consequences if not dealt with promptly.

## Case presentations (Table 1)

**Table 1:** Clinical and laboratory characteristics in five adults with parvovirus B19 manifestations in the central nervous system.

Case	Age/Sex	Neurological signs and symptoms	Associated findings	CSF bacterial culture	PCR PV-B19 Status	CSF analysis			Brain CT	Brain MRI	Treatment	Outcome
						WBC	Protein (mg/dL)	Glucose (mg/dL)				
1	74y/M	Tension headache and confusional state	N/A	Negative	Positive	0	107	57	Negative	PV and Lt occipital lesions	IVIG 30 g daily for 5 days	Improved
2	72y/M	Decreased level of consciousness	West-Nile Virus coinfection + ADEM	Negative	Positive	2300 (50% PN)	131	105	Atrophic brain + old infarct	Diffuse PV and white matter plaques	IVIG for 3 days + Dexamethasone and Epanutin	Improved
3	63y/M	Altered level of consciousness	Heart failure + Shock liver + Hepatic encephalopathy grade III	Negative	Positive	0	44	92	Negative	N/A	Supportive	Died
4	46y/F	Abnormal body movements/Seizures/LOC	Hb 8.1 g/dL WBC 16.3 x10^9^/L K^+^ 5.98 mmol/L	Negative	Positive	0	34	62	Bilateral basal ganglia hyperdensity	N/A	Levetiracetam phenytoin	Improved
5	46y/F	Abnormal bilateral jerky movements + Impaired awareness	MRSE (blood) *A. Baumannii* (sputum) *Candida* (urine)	Negative	Positive	0	47	273	Negative	Normal	IVIG 60 g for 2 days + Methylpridnisolone + Norepinephrine	Died

Abbreviations: M: Male; F: Female; LOC: Loss of consciousness; N/A: Not available; ADEM: Acute Disseminated Encephalomyelopathy; MRSE: Methicillin-resistant *S. epidermidis*; Lt: Left; PV: Periventricular.

### Case 1

A 74-year-old male with a medical history of type 2 diabetes mellitus (HbA1c 8.2%) and coronary artery disease presented complaining of new-onset neurological symptoms.

The patient was in his usual state of health until one day prior to admission when he started complaining of a tension headache at work, followed by progressive confusion and disorientation to time, place, and person.

The patient denied any fever or other systemic symptoms, on examination the patient was conscious but confused and not oriented to time, person and place. He was hemodynamically stable with no evidence of focal neurological deficits or meningeal irritation.

He was admitted as a possible case of ischemic stroke and to rule out central nervous system (CNS) infection. On admission, he had a non-contrast CT scan of the brain, which showed no acute changes. Lumbar puncture (LP) was also performed and sent for CSF analysis and culture, and revealed elevated protein (107 mg/dl), normal glucose (57 mg/dl), zero white blood cells, and a negative bacterial culture. A multiplex CSF PCR panel (including enteroviruses, HSV, EBV, CMV and VZV) CSF PCR returned negative, while B19-PV returned positive. Brain MRI was also performed and revealed periventricular and left occipital lesions, raising suspicion for meningoencephalitis.

Alternative causes for meningoencephalitis were excluded through a negative multiplex CSF panel, negative blood and CSF bacterial cultures, and neuroimaging (CT) that showed no midline shift or features suggesting of demyelinating disease.

He was started on intravenous immunoglobulin (IVIG) 30 g daily for 5 days, after which he showed improvement in his clinical status.

### Case 2

A 72-year-old male with a medical history of type 2 diabetes (HbA1c 7.59%), ischemic heart disease, and chronic urinary incontinence attributed to a prior ischemic stroke, presented with a decreased level of consciousness. The patient reported a fever reaching 39°C two days before admission, where he was evaluated in the ER and discharged with levofloxacin. The day after, his son observed progressive confusion and disorientation, and due to clinical deterioration, he was brought back to the hospital for further evaluation. Notably, the family reported recent close contact with a cousin who had been admitted to the ICU with encephalitis of an unconfirmed etiology.

He was isolated and admitted as a case of Meningoencephalitis. On examination, the patient looked ill, disoriented, and barely talked with confused speech. The neurological examination revealed tremors and bradykinesia; his GCS score was 11/15.

He was hemodynamically stable with an unremarkable chest X-ray and urine analysis. Brain CT was performed and showed an atrophic brain with old stroke infarcts.

Lumbar puncture was performed and revealed CSF WBC 2300 (50% Neutrophils), glucose 105 mg/dl, and Protein 131. However, CSF culture and viral PCR were initially negative. The patient underwent Brain MRI imaging and revealed Diffuse Periventricular and White matter Plaques, suggestive of small vessel ischemic changes.

He was started on IVIG for 3 days and showed minimal clinical improvement. A repeat CSF PCR test subsequently returned positive for B19-PV. On day six, serum West Nile virus IgM also tested positive.

MRI was repeated with contrast and confirmed widespread mild atrophic brain changes with a background of extensive small vessel ischemic disease. Given the poor IVIG response and the dual viral positivity, treatment was escalated to include dexamethasone 8 mg intravenously three times daily (total 24 mg/day) along with a 1000 mg loading dose of intravenous phenytoin (epanutin) over 3 hours, followed by 100 mg three times later that day.

Following treatment, the patient demonstrated gradual improvement in interactiveness and orientation to place and time, with no focal neurological deficits, although he remained mildly somnolent. He was discharged on a tapering dexamethasone regimen of 1 mg/day for five days followed by 0.5 mg/day for an additional five days.

### Case 3

A 63-year-old male with hypertension, diastolic heart failure, chronic atrial fibrillation, and psychiatric illness controlled with antipsychotics, presented to the emergency room after he was found collapsed at his home.

On admission, the patient was tachycardic (102), hypertensive (160/90), and hypoxic. On examination, crackles were heard bilaterally. Initial laboratory tests revealed increased troponin levels and a creatinine level of 2 mg/dl, whereas a 12-lead ECG showed atrial fibrillation without any significant changes, for which he was admitted as a case of NSTEMI, AKI, and decompensated heart failure.

On day 1, the patient developed an altered level of consciousness. Examination revealed a distended abdomen and a GCS of 12/15. Laboratory studies were remarkable for severe liver injury (AST 2500 U/l/ALT 1500 U/l), INR 5.5, ammonia level of 190 μmol/l, and hypoalbuminemia (2.5 g/dl) consistent with acute liver failure and Grade 3 hepatic encephalopathy. Intestinal obstruction was ruled out by abdominal CT, and brain CT ruled out any acute changes. The patient was intubated to protect the airway and transferred to the ICU later on. He had a chest CT, which revealed pulmonary edema and signs of pneumonia.

The patient was successfully extubated on day 4; however, disorientation persisted. The patient experienced further deterioration on day 6, necessitating reintubation.

LP was done to investigate underlying infectious CNS diseases. On day 7, the patient suffered 2 episodes of sudden cardiac arrest, and despite the return of spontaneous circulation for a brief time, the patient expired on the third cardiac arrest.

CSF analysis results, which came out 3 days post-mortem, returned positive for B19-PV on PCR testing, with a protein value of 44, a glucose value of 92 mg/dl, and zero WBCs, suggesting a diagnosis of viral encephalitis that may have contributed to his clinical deterioration.

### Case 4

A 46-year-old female with a 24-year history of type 2 diabetes mellitus, hypertension, severe mitral regurgitation, and end-stage renal disease managed with regular hemodialysis, presented with new-onset abnormal body movements involving all four limbs. These episodes, lasting between 2 to 10 minutes each, were associated with stool incontinence, which started 2 days prior to admission. For the above-mentioned symptoms, she was clinically diagnosed with Parkinson's two days ago and was started on levodopa, which has worsened her symptoms. The patient denied recent travel or sick contacts. However, a previous admission six months prior to this admission was due to similar episodes, which was managed as uremic chorea.

Upon examination, the patient was in respiratory distress with an SPO_2_ of 88% on room air and was hypertensive with a blood pressure of 152/101 mmHg, heart rate 82 bpm, temperature 37.4°C. During evaluation, she experienced a tonic–clonic seizure that lasted for 2 minutes, associated with loss of consciousness and bilateral eye deviation to the right.

Laboratory testing revealed anemia (Hb 8.1 g/dl), leukocytosis (WBC 16.3 × 10^9^/l), hyperkalemia (K^+^ 5.98 mmol/l), and normal serum ammonia. Other parameters were unremarkable.

She was later admitted to the ICU with a provisional diagnosis of status epilepticus and a suspected metformin-associated lactic acidosis. During her ICU stay, metformin was held, and her treatment regimen was adjusted to include levetiracetam, phenytoin, and pantoprazole. Hemodialysis sessions continued as usual. A subsequent brain CT showed bilateral basal ganglia hypodensity. A lumbar puncture was subsequently performed, showing clear CSF with protein 34 mg/dl, zero WBC count, and glucose 62 mg/dl. However, CSF PCR was positive for B19-PV.

On day 9, the patient’s clinical condition had returned to her baseline state. She was therefore discharged with instructions to continue her regular hemodialysis schedule.

### Case 5

A 46-year-old female with poorly controlled type 2 diabetes mellitus presented to the emergency room due to a decreased level of consciousness for the last 2 hours. Earlier on, her family observed sudden-onset abnormal bilateral jerky movements of her upper limbs accompanied by impaired awareness, tongue biting, frothy oral secretions, and upward eye deviation. The patient was also on NSAIDs for the past week for headaches. Upon arrival at the emergency room, she developed a second seizure with urinary incontinence and had a large coffee-ground vomitus, prompting immediate intubation and sedation with propofol and fentanyl.

On presentation, she was ill-appearing, intubated, afebrile, with a GCS < 8, blood pressure 180/98 mmHg, heart rate 85 bpm, and SpO₂ 92% on mechanical ventilation. Neurological examination was limited due to intubation but revealed symmetric yet unreactive pupils, normal reflexes, and positive Babinski on the right side.

Initial laboratory findings showed a pH of 7.31, HCO₃ of 18.3, lactate of 2, and glucose of 630 mg/dl. BUN/Cr was 25/1.7, with a progressive rise to 78/2, leukocytosis (WBC 23.9 × 10^9^/l), CRP 400. Lumbar puncture revealed clear cerebrospinal fluid, with 0 white blood cells, 140 red blood cells, protein concentration of 47 mg/dl, and glucose level of 273 mg/dl. PCR testing was positive for B19-PV. Brain CT and MRI were normal. However, an EEG showed diffuse symmetrical delta slowing consistent with severe encephalopathy.

On day 2, she became hypotensive and febrile, and developed new-onset diffuse erythema along the right upper limb with a non-blanchable rash; a provisional diagnosis of toxic-shock syndrome was considered. Broad-spectrum antibiotics (vancomycin, meropenem, and clindamycin) were initiated, in addition to intravenous immunoglobulin (IVIG) 60 g daily for 2 days and methylprednisolone. Other supportive medications included norepinephrine, insulin, and nutritional support via nasogastric tube.

Her ICU course was complicated by persistent fevers, AKI, ARDS, and shock. Repeated cultures revealed MRSE (Methicillin-resistant *Staphylococcus epidermidis*) (blood), *Acinetobacter baumannii* (sputum), and *Candida* (urine). The patient has not improved despite wider antimicrobial coverage.

On day 20, the patient experienced a cardiopulmonary arrest; resuscitation efforts were unsuccessful, and she was pronounced dead.

## Discussion

In this case series, five adult B19-PV cases were diagnosed within a one-year period in Palestine Medical Complex center, drawing attention to their diverse demographic features and clinical manifestations, which complicated the investigative process in some cases; in two patients with severe comorbidities, the infection may have triggered a sequence of exacerbations that worsened their clinical instability, ultimately contributing to their death.

B19-PV infection and associated neurological manifestations have been reported in the literature. However, most cases are found in the pediatric population [4], with limited data available on the adult population and vague associated neurological manifestations. This series provides unprecedented and unforeseen neurological findings that were later found to be due to B19-PV.

B19-PV infection typically begins with flu-like symptoms that persist for a duration of one or two weeks, which is also called phase 1, after which the body starts to produce antibodies to clear the infection and begin the second phase of the disease, which is characterized by arthralgias, arthritis, and skin rash [5, 6]. Unlike what is typical in the literature, our series of patients mainly presented with vague neurological manifestations that may mislead the physician into thinking of many other differential diagnoses.

Among the causes of encephalitis, which is a life-threatening inflammatory brain disorder, viral etiologies should be seriously considered, with other less likely causes, such as fungal, bacterial, and autoimmune conditions [7]. Our series underscores the need for clinical awareness of keeping B19-PV in the differentials when coming across such mysterious presentations in the adult population.

According to Sharma et al., in their review of more than 3000 patients with encephalitis, there was a pooled prevalence of 3% of patients within a subset of encephalitis, with studies using serological testing methods showing lower rates of infection in comparison to molecular assays, which could be explained partly due to the sensitivity and specificity rates [8]. The included studies presented notable variability due to differences in patient populations and geographic settings, presumably exploited by regional epidemiology, healthcare infrastructure, and diagnostic criteria for encephalitis [8].

Serum PCR, CSF PCR, and serology should be integrated in the diagnosis of B19-PV in cases with neurological manifestations, given the wide spectrum of CNS and PNS involvement [3]. According to Douvoyianni et al., in a review of 81 neurological cases, including encephalitis, meningitis, stroke, and peripheral neuropathy, B19-PV DNA was detected in 81% of CSF samples and 85% of serums, while only 33% had positive IgM in CSF samples, highlighting PCR’s critical role. Therefore, CSF and serum PCR should be the diagnostic cornerstone in the presence of neurological symptoms due to their high sensitivity, as serology alone might miss B19-PV in early or atypical presentations [3].

On the other hand, analysis of CSF cell counts, protein, and glucose in addition to neuroimaging findings, lacks diagnostic utility when it comes to B19-PV-associated encephalitis, due to the wide variety of these parameters with no consistent pattern [9].

Since no antiviral agents targeting B19-PV exist, immunomodulation remains the main treatment for B19-PV-associated neurological symptoms. IVIG and/or steroids were given to 16 patients in a case series conducted by Barah et al. [9]. Despite the clinical improvement of 12 cases, 12 other cases have also shown similar improvements without receiving either drug, suggesting inadvertent association [9]. A combination of both IVIG and corticosteroids was assigned to a total of 8 patients, which later showed clinical improvement, making it impossible to determine the individual efficacy of either agent [9].

Unlike the classical biphasic presentation described in most literature, which describes B19-PV manifestations that mainly consist of flu-like illness, followed by rash and arthralgia [5, 6]. Our case series uniquely reports five adult patients presenting with a diverse range of symptoms that made B19-PV an unfavourable diagnosis at their admission. Initial neurological symptoms, including confusion, altered consciousness, seizures, and encephalopathy, have dominated. These atypical manifestations initially led physicians to more common diagnoses such as ischemic stroke, hepatic and uremic encephalopathies, or even toxic metabolic syndromes, delaying the consideration of B19-PV or even other viral etiologies.

In all five cases, B19-PV was confirmed through CSF PCR after excluding more common infectious, autoimmune, or metabolic causes. CSF parameters such as white blood cell count, protein, and glucose were nonspecific or even normal in some cases, consistent with findings in the literature that points to their poor diagnostic value [9].

It is noteworthy that many of our patients had significant comorbidities, which likely masked the underlying B19-PV infection and clouded the clinical picture, delaying both diagnosis and treatment. Among our five cases, treatment strategies varied between our cases. Cases 2 and 5 were treated with a combination of IVIG and corticosteroids; case 2 demonstrated marked improvement, while case 5 succumbed to the illness. Case 1 received IVIG alone and recovered well. Interestingly, cases 3 and 4 received neither IVIG nor corticosteroids; their outcomes diverged, with case 3 resulting in death and case 4 achieving recovery.

It is important to note that detection of B19-PV DNA in CSF is not in itself pathognomonic for active CNS infection. Persistence of B19-PV in various tissues such as bone marrow, endothelium, and even the brain is not unusual [10]. Thus, PCR positivity alone does not confirm causation, as viral DNA may be present due to persistent low-level infection or passive entry across the blood–brain barrier in vulnerable individuals. In our cases, the lack of pleocytosis or significant protein elevation in CSF, together with normal neuroimaging in several patients, raise the possibility of an immune-mediated or post-infectious process rather than direct viral encephalitis. This interpretation is supported by literature, where Khawaja et al. in their review noted that many B19-PV-related CNS cases showed nonspecific CSF findings along with unremarkable neuroimaging [11]. Moreover, the positive response to immunotherapy in our cases raises the possibility of a post-infectious or immune-mediated neurological dysfunction. Therefore, cautious interpretation of PCR positivity for B19-PV is required to avoid the over-attribution of neurological symptoms to B19-PV when it may be a coincidental or a triggering factor rather than the sole pathogen.

## Conclusion

This case series highlights the critical need to consider Parvovirus B19 as a differential diagnosis in adult patients presenting with unexplained neurological symptoms. The variability in clinical presentations and often nonspecific laboratory findings can delay diagnosis, particularly in individuals with various comorbidities. Prompt recognition through CSF PCR testing is essential. Given the absence of targeted antivirals, immunomodulatory therapy may offer benefit, though further studies are needed to establish optimal management strategies in adult populations.
